# Mechanical tension and spontaneous muscle twitching precede the formation of cross-striated muscle *in vivo*

**DOI:** 10.1242/dev.140723

**Published:** 2017-04-01

**Authors:** Manuela Weitkunat, Martina Brasse, Andreas R. Bausch, Frank Schnorrer

**Affiliations:** 1 Muscle Dynamics Group, Max Planck Institute of Biochemistry, Am Klopferspitz 18, Martinsried 82152, Germany; 2 Lehrstuhl für Biophysik E27, Technische Universität München, James-Franck-Straße 1, Garching 85748, Germany; 3 Developmental Biology Institute of Marseille (IBDM), CNRS, UMR 7288, Aix-Marseille Université, Case 907, Parc Scientifique de Luminy, Marseille 13288, France

**Keywords:** *Drosophila*, Muscle, Tension, Myofibrillogenesis, Sarcomere, Self-organization

## Abstract

Muscle forces are produced by repeated stereotypical actomyosin units called sarcomeres. Sarcomeres are chained into linear myofibrils spanning the entire muscle fiber. In mammalian body muscles, myofibrils are aligned laterally, resulting in their typical cross-striated morphology. Despite this detailed textbook knowledge about the adult muscle structure, it is still unclear how cross-striated myofibrils are built *in vivo*. Here, we investigate the morphogenesis of *Drosophila* abdominal muscles and establish them as an *in vivo* model for cross-striated muscle development. By performing live imaging, we find that long immature myofibrils lacking a periodic actomyosin pattern are built simultaneously in the entire muscle fiber and then align laterally to give mature cross-striated myofibrils. Interestingly, laser micro-lesion experiments demonstrate that mechanical tension precedes the formation of the immature myofibrils. Moreover, these immature myofibrils do generate spontaneous Ca^2+^-dependent contractions *in vivo*, which, when chemically blocked, result in cross-striation defects. Taken together, these results suggest a myofibrillogenesis model in which mechanical tension and spontaneous muscle twitching synchronize the simultaneous self-organization of different sarcomeric protein complexes to build highly regular cross-striated myofibrils spanning the length of large muscle fibers.

## Abstract (precis)

**Summary:** In *Drosophila*, immature myofibrils are built simultaneously across an entire muscle fiber, and then self-organize in a manner dependent on spontaneous contractions and mechanical tension.

## INTRODUCTION

The muscular system is the major force-producing tissue of animals. In particular, the skeletal muscles enable precise body movements of invertebrates and vertebrates. For these accurate movements, each muscle must be properly connected to the skeleton. This is achieved by the attachment of both ends of the muscle fiber to tendons, which in turn connect to the skeleton. In large animals, it is often hundreds of fibers that are packed into muscle fiber bundles that run parallel to the long axis of the muscle. Thus, muscle is a highly polar tissue, which harbors a defined contraction axis between both tendon attachments (Hill and Olson, 2012).

The sarcomere is the contractile unit of each muscle fiber (Clark et al., 2002; Gautel and Djinovic-Carugo, 2016). Each sarcomere is symmetrically organized between two Z-discs, which cross-link antiparallel polar actin filaments, also called thin filaments. The centrally located thick filaments comprise bipolar myosin filaments. These thick filaments are permanently connected to the neighboring Z-discs by connecting filaments, largely formed by the gigantic protein titin (Gautel, 2011; Tskhovrebova and Trinick, 2003). This results in a stereotypical length for each sarcomere that is characteristic for the muscle type, ranging from ∼3.0 to 3.4 µm in relaxed human skeletal muscle *in vivo* (Ehler and Gautel, 2008; Llewellyn et al., 2008). As individual muscle fibers can be several centimeters long, it is necessary for hundreds, and often thousands, of sarcomeres to assemble into long chains called myofibrils during muscle development (Hill and Olson, 2012; Sanger et al., 2010).

Despite detailed textbook knowledge about mature sarcomere and myofibril architecture, our understanding of myofibril and sarcomere formation during muscle development is still limited. A proposed ‘ruler’ hypothesis suggests that titin, which spans from the Z-disc to M-line across half a sarcomere in mammalian muscle, sets sarcomere length (Fürst et al., 1988; Tskhovrebova and Trinick, 2003; Tskhovrebova et al., 2015; Whiting et al., 1989). However, it is unclear how such a ruler defines the characteristic sarcomere length of the different muscle types (Gokhin and Fowler, 2013). The ruler hypothesis also does not seem to be applicable to insect muscle, as individual insect titin homologs are too short to span across half a sarcomere. Nevertheless, insect sarcomere sizes are set as precisely as in vertebrates (Bullard et al., 2005; Tskhovrebova and Trinick, 2012). Likewise, it is debated how a large number of sarcomeres assemble into linear myofibrils. Different models propose that either short and irregular premyofibrils slowly mature into regular myofibrils by exchanging nonmuscle myosin II for muscle myosin II (Rhee et al., 1994; Sanger et al., 2010; Sparrow and Schöck, 2009) or, alternatively, that thin and thick filaments assemble more independently and subsequently interdigitate (Ehler et al., 1999; Holtzer et al., 1997; Rui et al., 2010). Data supporting these models were often acquired *in vitro* by analyzing cardiomyocytes or myotubes adhering to a Petri dish. This contrasts with the *in vivo* situation, in which both defined muscle fiber ends attach to tendons and thus set the polarity and contraction axis of the muscle fiber. Hence, it is important to study myofibrillogenesis using an *in vivo* model.

*In vivo*, vertebrate skeletal muscles have a typical cross-striated appearance (Hill and Olson, 2012), which is essential for the mechanism of muscle contraction (Huxley and Niedergerke, 1954; Huxley and Hanson, 1954). These cross-striations are formed by a regular lateral alignment of the individual myofibrils. During the formation of the aligned structure, Z-bands grow significantly in width (Sanger et al., 2010) and neighboring Z-discs might be linked by intermediate filaments (Gautel and Djinovic-Carugo, 2016). It has been found that even mature Z-discs dynamically exchange a number of Z-disc components with the cytoplasmic pool (Wang et al., 2005). This may contribute to the Z-disc growth and potentially to their gradual lateral alignment, resulting in the cross-striations of the muscle. However, the exact molecular mechanism of cross-striation formation *in vivo* remains elusive.

Recently, we have investigated myofibrillogenesis *in vivo* using the *Drosophila* indirect flight muscle model (Weitkunat et al., 2014). We found that after myotubes have attached to tendons, myofibrils assemble simultaneously throughout the entire myofiber. This results in continuous immature myofibrils that span across the entire 200 µm long muscle fiber, suggesting a self-organization mechanism for actin and myosin filaments, together with titin complexes. Importantly, myofibril formation is preceded by a build-up of mechanical tension within the flight muscle-tendon system, and if tension build-up is blocked or tension is released, myofibrillogenesis is severely compromised. This led to an extended model of myofibrillogenesis, which proposed that tension is an essential coordinator for myofibrillar self-organization in the flight muscles (Lemke and Schnorrer, 2016; Weitkunat et al., 2014). Tension and myosin contractility are also components of theoretical models aiming at predicting the dynamics of sarcomere assembly (Friedrich et al., 2012; Yoshinaga et al., 2010). However, the *in vivo* presence of tension was thus far only detected in indirect flight muscles of *Drosophila*, which display a specialized fibrillar organization of their myofibrils that enables fast contraction cycles, but lack the typical cross-striated pattern of vertebrate skeletal muscles (Josephson, 2006; Schönbauer et al., 2011; Weitkunat et al., 2014).

Here, we set out to investigate myofibrillogenesis and tension formation in the *Drosophila* adult abdominal muscles, which are cross-striated and synchronously contracting muscles and thus resemble vertebrate skeletal muscles. By performing *in vivo* imaging, we detect simultaneous myofibril assembly in these muscles and find that mechanical tension is not only present before but also during myofibril assembly. Remarkably, immature myofibrils, lacking an obvious periodic pattern, are already contractile when stimulated by Ca^2+^ influx, suggesting a sarcomere-like organization of their components at this early stage of development. Importantly, we find that the conversion of immature myofibrils to cross-striated myofibrils coincides with a strong increase of spontaneous muscle twitching, which is required to efficiently form cross-striations. Taken together, these results imply that there is a general role for mechanical tension and Ca^2+^-dependent spontaneous twitching in coordinating actomyosin self-organization to build regular cross-striated muscle fibers *in vivo*.

## RESULTS

### Abdominal muscle morphogenesis – an overview

**Fig. 1.**
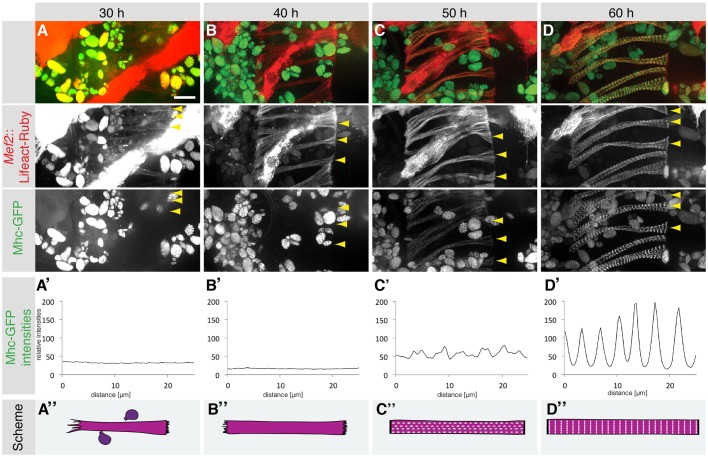
**Simultaneous sarcomerogenesis in *Drosophila* abdominal body muscles.** (A-D) Images of developing dorsal abdominal muscles (arrowheads) expressing Lifeact-Ruby (red) and Mhc-GFP (green) at 30 h (A), 40 h (B), 50 h (C) and 60 h (D) APF from a spinning disc confocal movie (Movie 1). (A′-D′) Relative Mhc-GFP intensities from representative longitudinal lines drawn within an abdominal dorsal muscle at the respective time points; Mhc-GFP expression appears between 40 h and 50 h APF simultaneously across the muscle fiber (B′,C′). (A″-D″) Schemata of developing dorsal abdominal muscles, Mhc-GFP is indicated in white. Scale bar: 25 µm.

*Drosophila* abdominal muscles form by fusion of adult myoblasts to myotubes at ∼24 h after puparium formation (APF) (Currie and Bate, 1991; Dutta et al., 2004; Krzemien et al., 2012; Weitkunat and Schnorrer, 2014). To analyze the development of the contractile apparatus *in vivo*, we imaged abdominal dorsal muscle development in intact pupae. We labeled the actin cytoskeleton with Lifeact-Ruby (Hatan et al., 2011) and labeled muscle myosin heavy chain (Mhc) by using a GFP trap within the endogenous *Mhc* gene (Clyne et al., 2003). At 30 h APF, the dorsal abdominal myotubes elongate along the anterior-posterior axis, forming dynamic leading edges at both myotube tips. Filopodia at these tips point to the direction of elongation (Movie 1; Fig. 1A). The filopodia at the posterior leading edge are less pronounced, suggesting that the posterior myotube tip is already in close contact with its future epidermal tendon cells (Krzemien et al., 2012). Filopodia dynamics gradually reduces until 40 h APF, suggesting that myotube-tendon attachment is also initiated at the anterior myotube tip (Movie 1; Fig. 1B). During this period Mhc-GFP is not yet detectable in the myotube and no obvious periodic actin pattern is found within the elongating myotubes (Fig. 1A,B).

Shortly before 50 h APF, Mhc protein becomes detectable and localizes in a periodic pattern throughout the myotube. Simultaneously with myosin, actin is also recruited into a similar periodic pattern (Movie 1; Fig. 1C). Initially, both patterns are irregular; however, they refine until 60 h APF, to form two distinct periodic patterns along the entire contraction axis of the myofiber (Movie 1; Fig. 1D). Taken together, these data suggest that actin is assembled into a periodic pattern when muscle myosin is expressed at significant levels, as detectable by live imaging. Interestingly, this periodic assembly occurs mostly simultaneously throughout the entire length of the myofiber, suggesting a self-organization mechanism for actin and myosin filaments.

### Abdominal muscle attachment

Studies in flight muscles have suggested that muscle attachment is required for myofibrillogenesis (Weitkunat et al., 2014). In order to investigate myotube attachment of abdominal muscles before and during myofibrillogenesis in detail, we fixed pupae and stained them for the bona fide attachment marker βPS-Integrin (also known as Myospheroid) (Brown et al., 2000; Leptin et al., 1989) at different developmental stages. In accordance with the live imaging, βPS-Integrin first concentrates at the posterior tips of the myotubes at 36 h APF, with little integrin present at the anterior tips (Fig. S1A,A′). However, anterior myotube tips are in close proximity to the overlaying epidermis and are therefore likely to form dynamic contacts with the epidermis at 36 h APF (Fig. S1A″). At 40 h APF, more βPS-integrin is present at the anterior myotube tips, suggesting that the myotube-epithelial tendon contacts are stabilized (Fig. S1B-B″). At 46 h APF, filopodia have largely disappeared from the myotube tips and more βPS-Integrin is localized at the tips, suggesting that the muscle-epithelial tendon contacts have further matured (Fig. S1C,C′). Interestingly, we detected epithelial cell extensions from 40 h onwards (Fig. S1B″,C″); these are similar to the tendon cell extensions produced during flight muscle morphogenesis when mechanical tension is built up (Weitkunat et al., 2014). At 52 h APF, even more βPS-Integrin is localized at the muscle fiber tips, where it remains until 72 h APF. During this phase, the myofibers continue to grow in length, despite remaining stably attached to their epithelial tendons (Fig. S1D-F). Taken together, these data substantiate the idea that abdominal myotubes begin to stably attach to tendon precursors at 40 h APF and build periodic myofibrils after 46 h APF.

### Myofibrillogenesis of cross-striated muscle

**Fig. 2.**
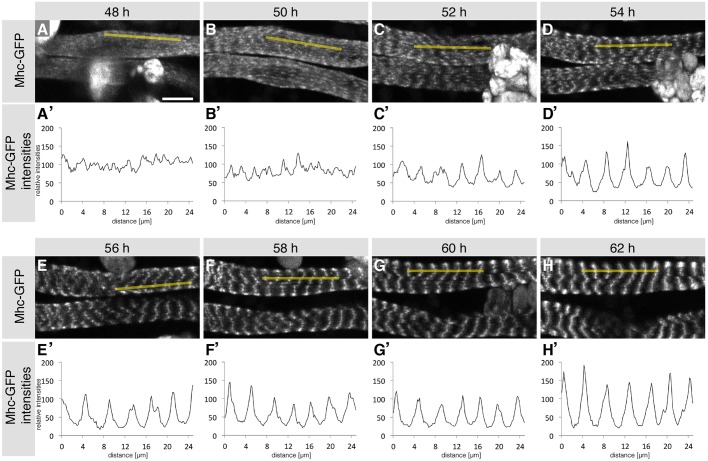
**Formation of cross-striated abdominal body muscles from live imaging experiments.** (A-H) Images of two developing dorsal abdominal muscles expressing Mhc-GFP at 48 h (A), 50 h (B), 52 h (C), 54 h (D), 56 h (E), 58 h (F), 60 h (G) and 62 h (H) APF from a multi-photon movie (Movie 2). (A′-H′) Contrast-adjusted Mhc-GFP intensities within one abdominal dorsal muscle (yellow lines in A-H) at the respective time points. Note the simultaneous appearance of Mhc-GFP periodicity from 50-52 h onwards and its lateral alignment. Scale bar: 10 µm.

In order to investigate the dynamics of cross-striated myofibrillogenesis at a high spatial resolution, we imaged intact pupae expressing Mhc-GFP from 48 h APF using multi-photon microscopy. This enabled us to follow individual muscle fibers *in vivo* over many hours of development. At 48 h APF, Mhc-GFP is present at low levels, localizing in a speckled pattern without obvious periodicity along the long axis of the muscle (Movie 2; Fig. 2A). These Mhc-GFP speckles become brighter and more organized by 50 h APF, building a defined periodic pattern along the entire muscle fiber by 52 h APF (Movie 2; Fig. 2B,C). Moreover, the periodic Mhc-GFP signal can be seen to align laterally to build the typical striated pattern, which becomes more refined over time (Movie 2; Fig. 2B-H). Importantly, the periodic Mhc-GFP pattern forms simultaneously along the future contraction axis of the muscle and the cross-striations also mostly appear at the same time throughout the entire muscle fiber, again suggesting a self-organization mechanism for the individual components to build the observed regular pattern.

**Fig. 3.**
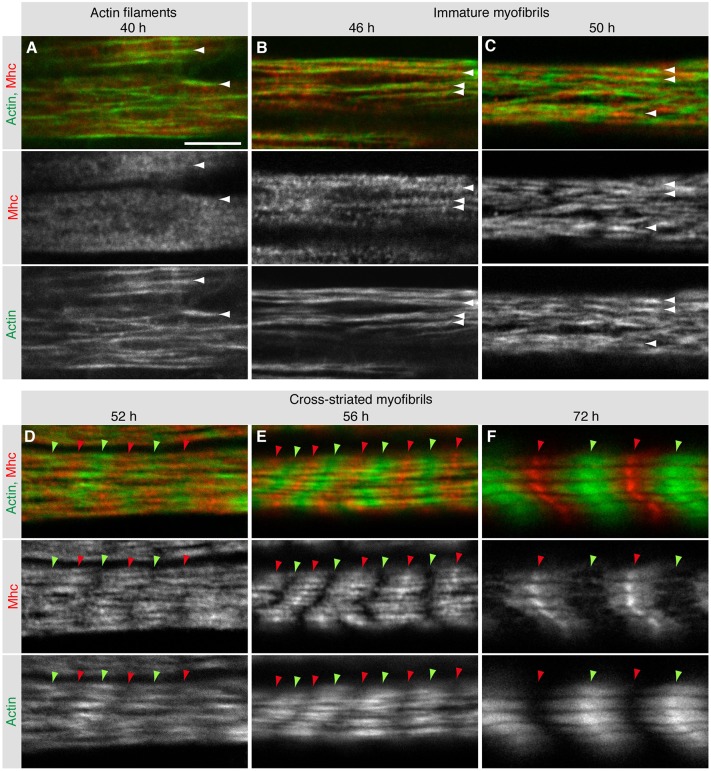
**Myofibril assembly of abdominal body muscles.** (A-F) Images of dissected wild-type abdomen at 40 h (A), 46 h (B), 50 h (C), 52 h (D), 56 h (E) and 72 h (F) APF. Actin (green) and Mhc (red) were labeled by phalloidin and anti-Mhc antibodies, respectively. Actin filaments are visible at 40 h (A, white arrowheads). Mhc is recruited to immature myofibrils (B,C, white arrowheads) in speckles at 46 h and in lines at 50 h APF. Both Actin and Mhc organize into striated patterns that align laterally and refine from 52 h-72 h APF (D-F, red and green arrowheads). Scale bar: 5 µm.

Next, we explored the relationship of actin and myosin filaments – the two major myofibril components – during myofibril assembly at high resolution by using images from fixed pupae. We used anti-Mhc antibodies and phalloidin to visualize Mhc and Actin, respectively. While the Mhc-GFP trap line only labels particular Mhc isoforms (Clyne et al., 2003; Orfanos and Sparrow, 2013), the antibody should label most Mhc isoforms, allowing a better visualization of the thick filaments. Phalloidin staining showed that actin filaments are present at 40 h APF. These actin filaments display an obvious polar orientation along the long myotube axis; however, they are still rather short and discontinuous. Importantly, the low levels of Mhc that are detectable by antibodies at 40 h APF reveal a speckled Mhc pattern throughout the myotube, without an obvious enrichment on actin filaments (Fig. 3A). This pattern changes until 46 h APF, when Mhc levels have increased and Mhc speckles are recruited onto the actin filaments, which themselves appear longer and more continuous (Fig. 3B). Although Mhc is still present in small speckles without a periodic pattern, we call these actin-myosin structures present at 46 h APF immature myofibrils.

Consistent with the live imaging results, Mhc expression increases further until 50 h APF when Mhc assembles into a periodic pattern that alternates with the actin pattern (Fig. 3C). As observed in the Mhc-GFP movies, the Mhc filament pattern is not yet laterally aligned at this stage. However, this changes rapidly, and cross-striated myofibrils with a prominent lateral alignment of actin and myosin filaments are detectable at 52 h APF (Fig. 3D). Consistent with our live imaging data, these striations further refine during the next few hours of development, resulting in distinct but overlapping actin and myosin filaments, which are laterally aligned by 56 and 72 h (Fig. 3E,F). Taken together, these data show a gradual maturation of the myofibrils throughout the muscle fiber and suggest that actin and myosin filaments self-organize to form cross-striated myofibrils.

### Mechanical tension precedes myofibrillogenesis

**Fig. 4.**
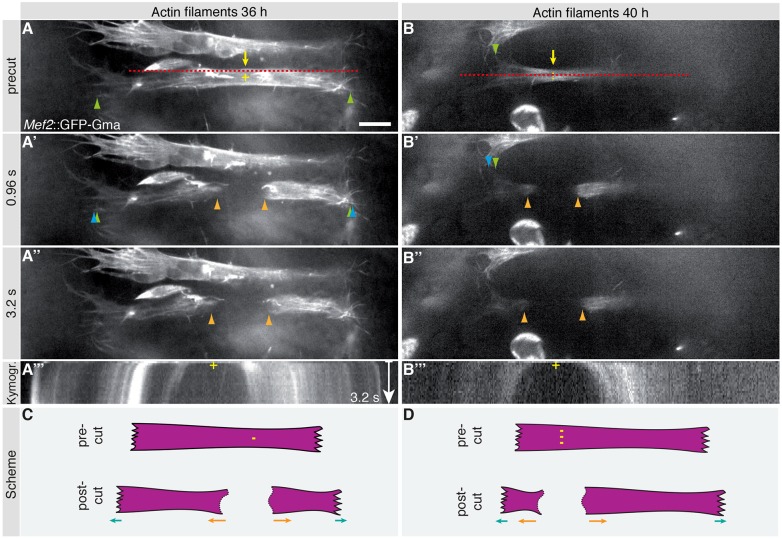
**Abdominal body muscles develop under mechanical tension.** (A-B″) Time points from spinning disc confocal movies of myotubes labeled by *Mef2-GAL4*, *UAS-GFP-Gma* at 36 h and 40 h APF before (A,B) and after (A′,A″,B′,B″) complete myotube severing using laser cutting (Movies 3, 4). Newly created myotube ends (orange arrowheads) move away from the cutting site (marked by ‘+’ in A,B). Anterior and posterior myotube ends move outwards; compare pre-cut (green arrowheads in A,B) with post-cut (blue arrowheads in A′, B′) ends. (A‴,B‴) Kymographs of Movies 3 and 4 displaying intensities at the red lines indicated in A and B. (C,D) Schemata of the laser cuts; myotube movement after laser severing is indicated by arrows. Scale bar: 10 µm.

In the non-cross-striated *Drosophila* flight muscles, we have previously demonstrated that mechanical tension precedes the formation of myofibrils. However, we had not been able to measure tension during the myofibril assembly or myofibril maturation itself (Weitkunat et al., 2014). It also remained unclear whether tension build-up also generally precedes myofibril formation in cross-striated muscle types. To investigate tension formation before and during myofibrillogenesis of cross-striated muscles, we performed laser lesion experiments using a pulsed UV laser (Mayer et al., 2010) to cut within abdominal myotubes at 36 h and 40 h APF. When performing a large lesion, to cut the myotube entirely, both myotube halves recoil significantly within the first second after the cut (Movies 3, 4; Fig. 4). Additionally, the myotube ends move outwards after the cut, supporting the idea that the myotube has indeed made mechanical contacts with the overlaying epithelium during these stages and has built up mechanical tension across the muscle (Fig. 4A′,B′,C,D). A similar recoil is also detected after a smaller micro-lesion, which only partially severs the myotube (Movies 5, 6 and Fig. S2). These data demonstrate that mechanical tension is indeed present within the myotubes from 36 to 40 h APF, which is the stage before immature myofibrils are assembling. This suggests that mechanical tension generally precedes myofibril assembly in developing muscle, including cross-striated muscle types.

### Immature myofibrils are contractile

**Fig. 5.**
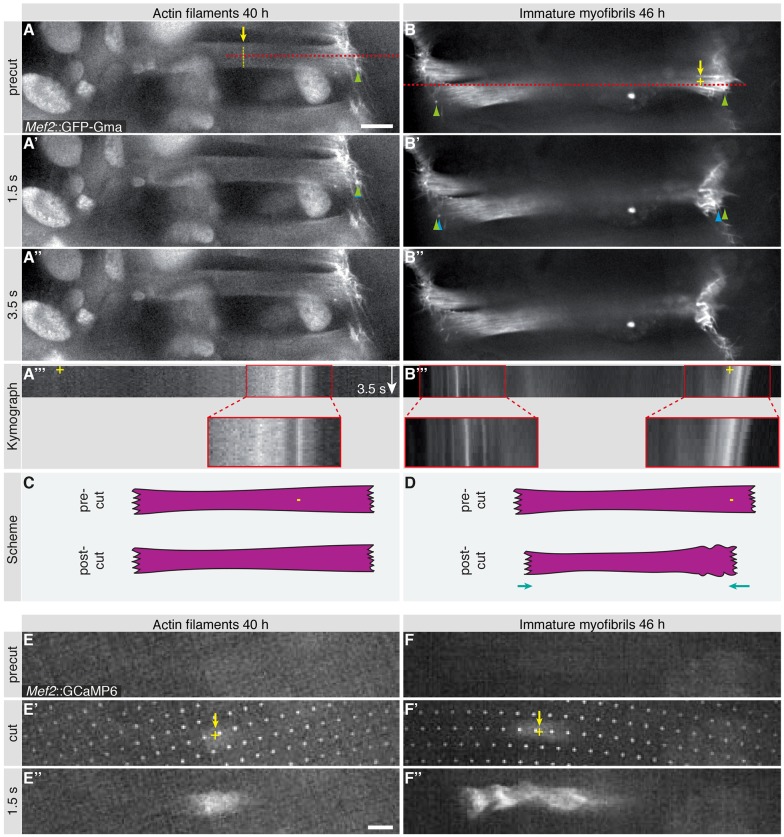
**Laser-induced myotube contractions during development.** (A-B″) Time points from spinning disc confocal movies of myotubes labeled by *Mef2-GAL4*, *UAS-GFP-Gma* at 40 h and 46 h before (A,B) and after (A′,A″,B′,B″) laser-induced nano-lesion (Movie 8). At 46 h APF, anterior and posterior myotube ends move inwards after nano-lesion indicating myotube contraction; compare pre-cut (green arrowheads, B) with post-cut (blue arrowheads, B′) ends. (A‴,B‴) Kymographs of Movie 7 displaying intensities at the red lines indicated in A and B. (C,D) Schemata of the laser cuts; myotube movement after nano-lesion is indicated with arrows. (E-F″) Ca^2+^ imaging of myotubes labeled with *Mef2-GAL4*; *UAS-GCaMP6* at 40 h and 46 h APF before (E,F), at (E′,F′, grid indicates the cut) and after (E″,F″) laser-induced nano-lesion (Movie 9). Both after nano-lesion at 40 h (E′,E″) and 46 h APF (F′,F″), a Ca^2+^ signal is visible in myotubes. Scale bars: 10 µm.

In order to investigate whether tension is also present at 46 h, when immature myofibrils have assembled, we performed the same micro-lesion experiments as above, leading to a surprising result – the injured myofiber starts to contract after the laser lesion (Movie 7 and Fig. S3). To explore this interesting result in more detail, we only induced a nano-lesion in the muscle, which does not result in a visible rupture. Such a nano-lesion has no effect on overall muscle morphology at 40 h APF (Movie 8; Fig. 5A,C). Strikingly, however, the nano-lesions induce muscle fiber contractions at 46 h APF, resulting in both fiber ends moving closer together, instead of further apart (Movie 8; Fig. 5B,D). As an influx in Ca^2+^ ions is the trigger for sarcomere contractions in mature muscles, we tested whether nano-lesions result in a cytoplasmic Ca^2+^ peak in the developing muscles. By applying the Ca^2+^ indicator GCaMP6 (Chen et al., 2013), we indeed detected a strong Ca^2+^ increase within the muscles following the nano-lesion, both at 40 h and 46 h APF (Movie 9; Fig. 5E,F). Supposedly, Ca^2+^ is released from laser-fragmented intracellular stores into the cytoplasm, where it triggers muscle fiber contraction at 46 h but not at 40 h APF. These data demonstrate that the immature myofibrils that have started to incorporate Mhc, but not the actin filaments present at 40 h APF, are capable of contracting upon release of Ca^2+^. Moreover, the entire muscle fiber must be mechanically coupled at 46 h APF as the fiber contraction is present at both muscle ends (Fig. 5B′,B″). These results are consistent with a self-organization of actin and myosin filaments into myofibrils across the entire muscle fiber.

### Myofibril contractility increases before striations appear

**Fig. 6.**
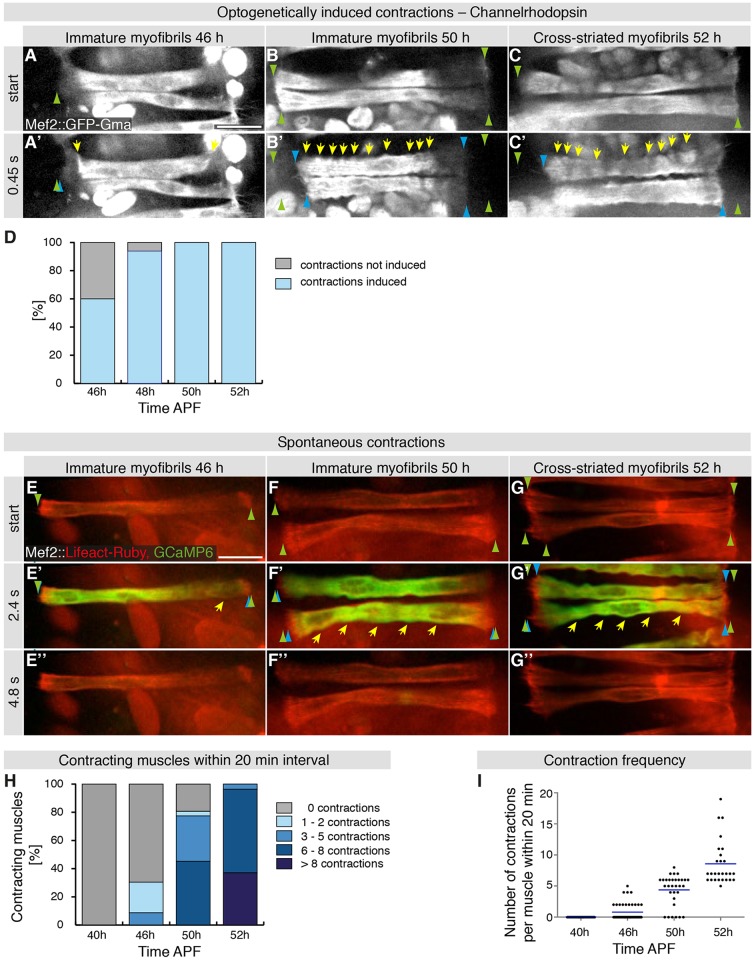
**Optogenetically induced and spontaneous myotube contractions during development.** (A-C′) Time points from spinning disc confocal movies of myotubes labeled by *Mef2-GAL4, UAS-GFP-Gma* and additionally expressing *UAS-Channelrhodopsin* at 46 h (A), 50 h (B) and 52 h (C) APF (Movie 10). Ca^2+^ influx and contractions are induced while imaging with 488 nm laser light. Bulges are marked by yellow arrows (A′,B′,C′) and myotube end movements with green and blue arrowheads. Note that myotube contractions increase at 50 h APF. (D) Quantification of myotube contractions; the number of contracting myotubes increases from 46 h to 50 h APF. Number of pupae: 8 at 46 h, 48 h and 50 h; 6 at 52 h. (E-G″) Time points from spinning disc confocal movies of myotubes labeled with *Mef2-Gal4*, *UAS-Lifeact-Ruby* and *UAS-GCaMP6* imaged for a 20 min interval at 46 h (E-E″), 50 h (F-F″) and 52 h (G-G″) APF. Ca^2+^ influx is visualized in green and muscles in red (Movie 11). Bulges are marked by yellow arrows (E′,F′,G′) and myotube end movements with green and blue arrowheads. Note that myotube contractions increase at 50 h APF. (H) Number of myotubes that contract within 20 min intervals at 40 h, 46 h, 50 h and 52 h APF. Number of muscles: 38 at 40 h; 46 at 46 h; 31 at 50 h; 27 at 52 h. (I) Contraction frequency during development. Each point represents number of contractions of one myotube within a 20 min interval. The mean contraction frequency (blue line) increases with time. Scale bars: 50 µm.

Since laser-induced nano-lesions could induce changes other than solely increasing Ca^2+^ ions, we aimed to increase cytoplasmic Ca^2+^ concentrations directly using optogenetics. We expressed the light-gated cation channel Channelrhodopsin (Boyden et al., 2005) in muscles and activated it with 488 nm light, the same wavelength used to image muscle morphology. Interestingly, upon channel activation at 46 h APF, we indeed observed small muscle contractions in ∼60% of the stimulated muscle fibers (Movie 10; Fig. 6A,D). Both, the intensity of the induced contractions and the number of contraction incidents increased with development, resulting in strong contractions along the entire muscle fiber in all stimulated muscles at 50 h or 52 h APF (Movie 10; Fig. 6B-D). These data show that a depolarization-induced Ca^2+^ peak efficiently induces myofiber contractions from 50 h APF onwards. Interestingly, this matches the developmental time period when immature myofibrils (50 h APF) transition to become cross-striated myofibrils (52 h APF).

### Spontaneous contractions precede cross-striations

Next, we asked whether contractions occur spontaneously in the muscles during this critical developmental period between 40 and 52 h APF. To address this question, we imaged developing muscles expressing Lifeact-Ruby and GCaMP6 with a high time resolution to monitor muscle morphology and cytoplasmic Ca^2+^ levels at the same time. We find that, at 40 h APF, muscles do not contract spontaneously (Fig. 6H,I). At 46 h APF, 30% of muscles do show small spontaneous contractions within a 20 min observation period. These contractions are always associated with a transient strong increase in cytoplasmic Ca^2+^ levels (Movie 11; Fig. 6E,H,I). Importantly, at 50 h APF most (81%) and at 52 h APF all imaged muscles strongly contract at least once within the 20 min observation period (Movie 11; Fig. 6F-I). The average contraction frequency increases during development from 0.8 contractions within 20 min at 46 h APF to 8.6 contractions within 20 min at 52 h APF (Fig. 6I). This demonstrates that spontaneous muscle twitching occurs frequently during the developmental period preceding the appearance of cross-striated myofibrils. It also shows that immature myofibrils at 50 h APF are already highly contractile. Taken together, these data strongly support the hypothesis that the periodic actomyosin arrays in the assembling myofibrils are mechanically coupled throughout the entire muscle fiber and are responsive to stimulatory Ca^2+^ signals.

### Spontaneous contractions contribute to cross-striation formation

In order to functionally investigate the role of the spontaneous contractions for cross-striation formation, we aimed to block the contractions from 46 h APF onwards and investigate the consequences for Mhc-GFP localization in the muscles. We tried to optogenetically block the contractions using Halorhodopsin (Fenno et al., 2011), but failed to do so reliably and continuously over several hours of muscle development (data not shown). As an alternative approach, we used Thapsigargin, a chemical inhibitor of the sarco/endoplasmic reticulum Ca^2+^-ATPase (SERCA), the main Ca^2+^ pump located in the membrane of the sarcoplasmatic reticulum (Treiman et al., 1998). To assess the potency of Thapsigargin, we injected it into the abdomen of pupae between 52 h and 53 h APF and imaged these at 55 h APF, a stage after which spontaneous contractions have been initiated (Fig. 6H,I). Indeed, we find that Thapsigargin is a potent blocker of these spontaneous contractions (Movie 12).

**Fig. 7.**
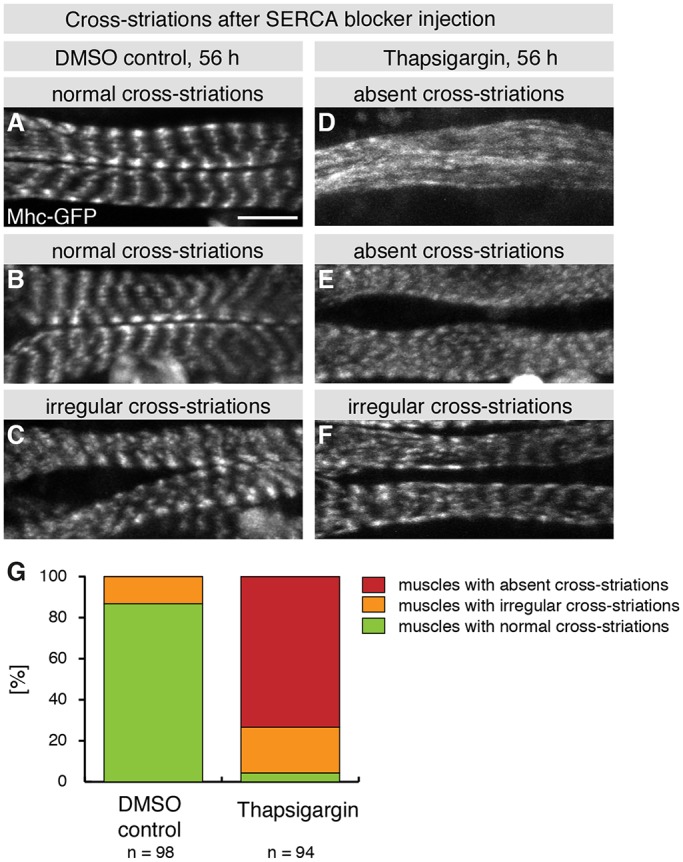
**Contractions contribute to cross-striation formation.** (A-F) Images of Mhc-GFP-expressing pupae, either injected with DMSO (A-C) or with Thapsigargin (D-F) at 46 h APF and taken at 56 h APF. Three representative examples of the phenotypic spectrum, ranging from normal to irregular and absent cross-striations, are shown for control and Thapsigargin injection. (G) Quantification of the cross-striation phenotypes of the injected pupae according to the phenotypic range shown in A-F. Scale bar: 10 µm.

To test the impact of the contractions on cross-striation formation, we injected Thapsigargin into pupae at 46 h APF, when the contractions normally begin to occur, incubated them for 10 h and imaged Mhc-GFP distribution at 56 h APF at high resolution using a multi-photon microscope. We find that 87% of the control-injected pupae show normal cross-striations at 56 h APF (Fig. 7A-C,G), whereas 73% of the Thapsigargin-injected pupae fail to build cross-striations in their abdominal muscles close to the injections site (Fig. 7D-G). These data demonstrate that Ca^2+^-induced contractions after 46 h APF are required to assemble regular cross-striations in *Drosophila* abdominal muscles.

## DISCUSSION

Myofibrils displaying a periodic sarcomere pattern are built during muscle development. Muscle fibers can be very long, more than 20 cm for a number of human muscles, while sarcomeres are below 4 µm in most animals (Burkholder and Lieber, 2001). Therefore, the precise periodic assembly of hundreds or often thousands of sarcomeres into long linear myofibrils is a challenging task. Our results demonstrate that muscles approach this task by first attaching both muscle fiber ends to tendon cells. When attachment is initiated, the actin cytoskeleton is polarized along the long axis of the muscle but does not develop periodic order at this stage. When muscle attachments have matured, a periodic actomyosin pattern assembles mostly simultaneously across the entire muscle fiber length, suggesting sarcomeric self-organization to build long continuous myofibrils. The concurrence of attachment maturation and myofibril self-organization is not only observed in body wall muscles, which resemble vertebrate skeletal muscles, but also in the specialized flight muscles (Weitkunat et al., 2014), strongly suggesting that myofibril self-organization is a general mechanism to assemble myofibrils within muscle fibers *in vivo*. The beauty of such a mechanism is that it always results in periodic myofibrils spanning across the entire muscle fiber, independently of the total fiber length. A similar periodic actomyosin self-organization has been predicted by theoretical models (Friedrich et al., 2012; Yoshinaga et al., 2010) and has also been found in nonmuscle cells, such as the stress fibers of cultured cells (Pellegrin and Mellor, 2007) and the peri-junctional actomyosin belts present in certain epithelial cell sheets *in vivo* (Ebrahim et al., 2013). Hence, simultaneous self-organization appears to be a general mechanism to create periodic actomyosin structures, with developing muscles being a particularly prominent example.

The synchrony of pattern formation suggests that the individual components are cooperating during the assembly process. We have previously shown that mechanical tension is required to build the highly regular myofibrils of the specialized flight muscles (Weitkunat et al., 2014). Here, we expanded these studies to the cross-striated body muscles of the adult fly and show that tension is not only present before but also during simultaneous myofibril assembly. Importantly, we found that immature myofibrils (i.e. myofibrils that had started to incorporate muscle myosin but that did not yet display a periodic pattern) already twitch in response to increased Ca^2+^ levels. This demonstrates that the individual components within immature myofibrils are already mechanically coupled along the fiber axis. The active contractions also suggest that myosin motors create forces that contribute to the tension build-up during myofibril assembly. This is supported by myosin inhibitor studies *in vitro* (Kagawa et al., 2006) and by the expression of motor-deficient myosin variants *in vivo* (Weitkunat et al., 2014), both of which result in severe myofibrillogenesis defects. The Ca^2+^-induced twitching of immature myofibrils also implies that the Ca^2+^-dependent troponin and tropomyosin machinery, which regulates mature muscle contractions (Ohtsuki and Morimoto, 2008), is co-assembling together with the periodic actomyosin pattern and is already controlling active myofibril twitching during development.

**Fig. 8.**
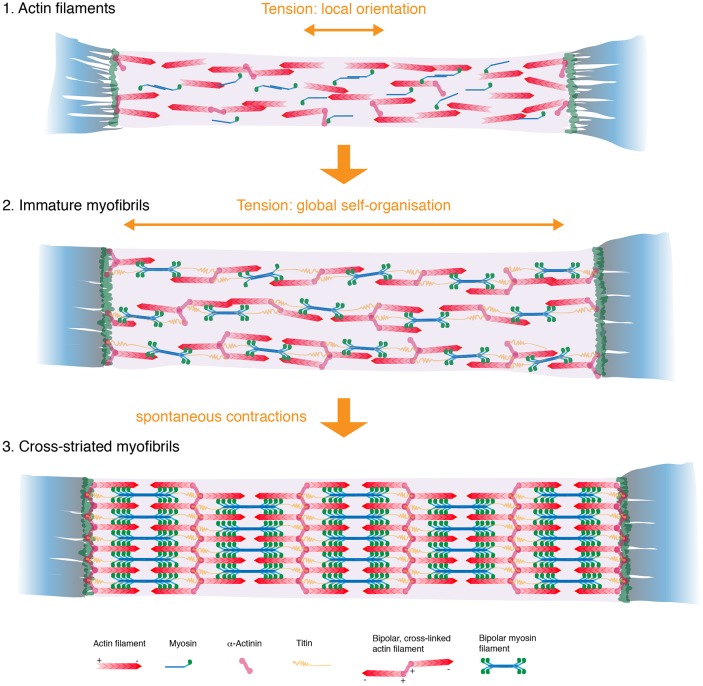
**Tension-driven model of myofibrillogenesis.** Locally, tension orients actin and myosin filaments along the axis of the muscle to assemble linear myofibrils (1). Globally, it coordinates the synchronous formation of periodic actomyosin filaments across the entire muscle fiber (2). Spontaneous muscle twitching contributes to the self-organization of perfectly ordered striated myofibrils (3). For further details, see the Discussion.

We have incorporated these data into an updated myofibrillogenesis model, which proposes two roles for mechanical tension, a local and a global one. Locally, tension can act as a molecular compass to orient individual myofibrillar components, like bipolar actin and myosin mini-filaments, along the long axis of the muscle. Thereby, it creates linear myofibrils with periodically arranged sarcomeres. Globally, tension can coordinate the self-organization process across the entire muscle fiber. This global coordination synchronizes the assembly process and results in balanced forces throughout the system. This synchrony appears analogous to phase transitions from unordered to more-ordered states, when tension is large enough, or molecularly speaking, when enough myosin has been recruited onto the myofibrils to pull cross-linked bipolar actin filaments into a periodic order (Fig. 8).

Such a tension-supported myofibrillogenesis model likely also applies to mammals. In the mammalian heart, myofibrils are anchored at specialized adherens junctions that mechanically couple myofibrils across cell membranes of neighboring cardiomyocytes (Perriard et al., 2003). If cardiomyocytes are grown individually in suspension and are therefore not mechanically coupled, effective myofibrillogenesis is blocked (Marino et al., 1987). Similarly, skeletal muscles that are defective in integrin function and thus cannot effectively generate tension, fail to assemble normal myofibrils during embryonic development in mice (Schwander et al., 2003). However, direct *in vivo* evidence for an instructive role of mechanical tension during myofibrillogenesis awaits live *in vivo* imaging of myofibril formation in developing mammalian muscle.

Mature mammalian heart or skeletal muscles, as well as *Drosophila* body wall muscles, are cross-striated. Formation of cross-striations requires the lateral alignment of neighboring myofibrils into a register, an essential process that is not well investigated in developing muscles *in vivo*. Both our live imaging and our immunohistochemistry data demonstrate that the transition from immature, non-aligned myofibrils to cross-striated myofibrils occurs simultaneously across the entire myofiber. This again strongly argues for a globally coupled system. Interestingly, the occurrence of the spontaneous muscle fiber contractions coincides with myofibril alignment. Myofiber contractions are detectable at 46 h APF *in vivo* and their frequency strongly increases until 50 h APF, shortly before regular actomyosin cross-striations are detected. Indeed, when the contractions are blocked by blocking Ca^2+^ cycling with the SERCA inhibitor Thapsigargin, the formation of cross-striations is severely impaired. Although it is difficult to rule out an indirect effect of potential endoplasmic reticulum (ER) stress induced by the SERCA block, these data strongly suggest that Ca^2+^-dependent actomyosin twitches refine the actomyosin periodicity and result in the efficient lateral alignment of neighboring myofibrils, an essential maturation step to build cross-striated muscle (Fig. 8).

A role for Ca^2+^-dependent twitches has also been suggested for mammalian myofibrillogenesis through *in vitro* experiments. Blocking membrane depolarization and spontaneous twitching in cultured rat myoblasts resulted in severe sarcomerogenesis defects (De Deyne, 2000). Conversely, electrically induced Ca^2+^ peaks could effectively promote sarcomere assembly in C2C12 cell-derived myotubes *in vitro* (Fujita et al., 2007). Furthermore, it has been shown that neuronal innervation, and thus spontaneous muscle twitching, results in increased cross-striations in cultured *Xenopus* myotubes (Kidokoro and Saito, 1988). Similar to the twitching we found in developing *Drosophila* muscles *in vivo*, the contractions present or induced in cell culture also resemble contractions of mature muscle because they require ryanodine receptor (RyR)-dependent Ca^2+^ cycling (Ferrari et al., 1998). Interestingly, either blocking the RyR *in vitro* (Harris et al., 2005) or knocking it out *in vivo* results in severe myofibrillogenesis defects, with RyR mutant mice having only small muscles that lack cross-striations (Barone et al., 1998; Takeshima et al., 1994). Taken together, these observations strongly suggest that Ca^2+^-dependent myofibril twitching is important for myofibril cross-striation formation during mammalian muscle morphogenesis. As mammalian muscle fibers are often at least one magnitude larger than *Drosophila* muscle fibers, tension-dependent self-organization is likely even more critical for the formation of regular cross-striated mammalian muscle. As muscle growth and muscle regeneration continues through human life, defects in tension-supported myofibril self-organization may result in severe myofibril disarrays and fatal myopathies (Clarke, 2008; Tajsharghi and Oldfors, 2013; Udd, 2008).

## MATERIALS AND METHODS

### Fly strains

All fly work, unless otherwise stated, was performed at 27°C to enhance GAL4 activity. Muscle-specific expression was achieved using *Mef2-GAL4* (Ranganayakulu et al., 1996). Abdominal muscles were labeled with *Mef2-GAL4*, *UAS-GFP-Gma* (Dutta et al., 2002), *UAS-Lifeact-Ruby* (Hatan et al., 2011), *UAS-Cherry-Gma* (Millard and Martin, 2008) or *Mhc-GFP (Mhc^Wee-P26^)* (Clyne et al., 2003). Ca^2+^ was imaged by using *UAS-GCaMP6f* (BL#42747, gift of Alex Mauss, Max Planck Institute of Neurobiology, Martinsried, Germany) (Akerboom et al., 2012) and muscles were depolarized with *UAS-Channelrhodopsin2-H134R-mCherry* (*UAS-ChR2-H134R*, gift of Alex Mauss) (Pulver et al., 2009).

### Fixed analysis of developing abdominal muscles

Staged wild-type pupae (*white^1118^*) were dissected as described previously (Weitkunat and Schnorrer, 2014). To relax the myotubes, the dissections were performed in cold relaxing solution followed by fixation in relaxing solution with 4% paraformaldehyde (PFA). After washing in PBS containing 0.3% Triton X-100 (PBST), dissected pupae were blocked for 30 min with normal goat serum (1:30), stained with primary antibodies overnight at 4°C and washed three times in PBST. Primary antibodies were: mouse anti-β-PS-Integrin (1:500; CF.6G11, DSHB), mouse anti-Mhc (1:100; Judith Saide, Department of Physiology and Biophysics, Boston University, MA). Secondary antibodies (at 1:500, Molecular Probes), Rhodamine-phalloidin (1:500) or phalloidin conjugated to Alexa Fluor 488 (1:500) (all from Molecular Probes) were added for 2 h at room temperature, followed by three washing steps in PBST, before samples were embedded in Vectashield. Images were acquired with a Zeiss LSM 780 and processed with Fiji (Schindelin et al., 2012) and Photoshop software.

### Time-lapse movies

GFP-expressing pupae were staged and a small opening was cut into the pupal case on the dorso-lateral side of the abdomen using sharp forceps and scissors. Pupae were transferred into a custom-made slide with a slit fitting the pupa and turned 20-30° resulting in abdominal myotubes facing up. The opening was covered with a thin layer of 86% glycerol and a coverslip to prevent evaporation. *Z*-stacks were acquired every 5 to 20 min with a multi-photon set up (LaVision) using a long distance 20× objective (NA=1.0, Zeiss) or spinning disc confocal microscope (Zeiss, Visitron) using a 40× long distance objective (NA=1.0, Zeiss). The microscope stage was heated to ∼27°C.

### Tension measurements

Muscle severing and imaging was performed on a custom-made nano-dissection device based on that presented in Colombelli et al. (2009), including a spinning-disc unit (CSU-X1, Yokogawa) with an Andor NEO sCMOS camera and a 63×1.20 NA water or a 63×1.40 NA oil objective (Leica Microsystems). Laser output was: 355 nm, 350 psec pulse duration, 72 kW peak power and 25 mW average power. Imaging was performed with the spinning disc unit and a COBOLT MLD™ 488 nm laser. Movies were taken at frame rates between 2 fps and 12.5 fps. Images and movies were processed with Fiji. Tension release in severed muscles was inferred from the responses of cut edges, structures along the muscle and attachment sites to severing. For Ca^2+^ imaging during laser-cutting, muscles were labeled using *Mef2-GAL4*,* UAS-Cherry-Gma* or *UAS-CD8-Cherry*, and Ca^2+^ was imaged through use of the GCaMPG6 maker expressed via *UAS-GCaMPG6f*. Pupae at respective time points were live imaged with a 561 nm laser (COBOLT Jive 50TM) to position the pupae on the nano-dissection device for the subsequent optical stimulation. Subsequently, the pupae were optically stimulated by the 355 nm laser (1 pulse) and imaged with the 488 nm laser.

### Quantification of spontaneous contractions

Muscles were labeled using *Mef-GAL4, UAS-Lifeact-Ruby* and Ca^2+^ was imaged by using *UAS-GCaMP6f*. Pupae of the respective age were prepared for live imaging and imaged for 20 min at 600 ms intervals on a spinning disc microscope. Contractions were counted manually. Intensity of the GCaMP6f signal was quantified using Fiji. Contractions per minute were calculated using Excel, and graphs were designed using Adobe Illustrator and Prism (GraphPad).

### Induction of contractions using Channelrhodopsin

*UAS-Channelrhodopsin2-H134R-mCherry* was expressed using *Mef2-GAL4* and muscles were labeled with *UAS-GFP-Gma*. Yeast paste containing 1 mM all-trans-retinal (Sigma) was mixed into the fly food containing the larvae one day before the pupae were staged for imaging. Pupae were then kept in the dark until imaging. Channelrhodopsin was activated by using the 488 nm laser; this wavelength was simultaneously used for GFP excitation, and 40 time points were imaged at 50 ms intervals on a spinning disc microscope. This was repeated eight times on the same pupa with 60 s breaks in-between repetitions. The second repeat was used for analysis.

### Pupal injections

Similar to for the time-lapse movies, a small opening was cut into the pupal case of 46 h APF *Mhc-GFP* pupae. A small amount of either DMSO or 2.5-5 mM Thapsigargin (Sigma) dissolved in DMSO was injected using a self-made glass needle and a FemtoJet injection system (Eppendorf). Injected pupae were transferred into custom-made imaging slides, put back into the incubator and imaged with a multi-photon microscope at high resolution at 56 h APF. Injections were usually performed into the left half of abdominal segment A2, and all visible dorsal longitudinal muscles in abdominal segments A2 and A3 were used to quantify the cross-striations.

For the initial tests of drug efficiency, *Mef-GAL4*, *UAS-Lifeact-Ruby* and *Mhc-GFP* pupae were similarly injected at 52-53 h APF and imaged to assess the spontaneous contractions at 55 h APF at 300 ms intervals on a spinning disc microscope.
